# Supplementary material for: Investigation of electromagnetic wave propagation characteristics across various frequencies in porous media of goaf
Source: PLoS One. 2024 Dec 18;19(12):e0315888. doi: 10.1371/journal.pone.0315888 (PMC11654933; doi:10.1371/journal.pone.0315888)
Supplement: Attachment — Submitted filename: Response to reviewers.docx [file pone.0315888.s004.docx]

Dear Editor:

Thank you for reviewing our manuscript and providing us with valuable feedback. We have carefully examined and revised all relevant sections of the manuscript, addressing each of the comments raised by you and the reviewers. Our specific responses are as follows:

**1.Completeness and Accuracy of References**

We have thoroughly reviewed the reference list to ensure its completeness and accuracy.

**2.Formatting of References**

We have revised the references to comply with the journal’s formatting guidelines. This includes correcting the details of author names, publication years, titles, journal names, volume numbers, and page ranges. All references have been double-checked to ensure there are no omissions or errors.

**3.Other Formatting Issues**

In accordance with the reviewers’ suggestions, we have further adjusted the overall formatting of the manuscript. This includes paragraph indentation, heading styles, font consistency, and line spacing to fully meet the journal’s requirements.

**4.Additional Notes**

All modifications made during this revision process have been highlighted in the manuscript and detailed in the response letter. We hope these revisions address your concerns. Should there be any further feedback or additional requirements, we would be more than happy to make the necessary changes.

Once again, we sincerely thank you and the reviewers for your diligent efforts and constructive feedback on our manuscript. Please feel free to contact us if there are any further questions or suggestions.

Best regards

Dr. Hui Zhuo on the behalf of the authors
